# Synthesis of High-Performance Aqueous Fluorescent Nanodispersions for Textile Printing—A Study of Influence of Moles Ratio on Fastness Properties

**DOI:** 10.3390/molecules26237075

**Published:** 2021-11-23

**Authors:** Shruthi Manjunath Shenava, J. V. Shanmukha Kumar, Rajkumar Ganugula, Mohammed Rafi Shaik, Rosa Busquets, Mohammad Rizwan Khan

**Affiliations:** 1 Aron Universal Limited, #25/1, 2nd Phase, Jigani Industrial Area, Jigani, Bangalore 560105, Karnataka, India; 2 Department of Chemistry, Koneru Lakshmaiah Education Foundation, Vaddeswaram, Guntur 522502, Andhra Pradesh, India; shanmukh_fed@kluniversity.in; 3 Department of Chemistry, College of Science, King Saud University, P.O. Box 2455, Riyadh 11451, Saudi Arabia; mrkhan@ksu.edu.sa; 4 School of Life Sciences, Pharmacy and Chemistry, Kingston University London, Penrhyn Road, Kingston-upon-Thames KT1 2EE, Surrey, UK; r.busquets@kingston.ac.uk

**Keywords:** fluorescent, nanodispersions, textile printing, moles ratio

## Abstract

Aqueous fluorescent dispersions containing dyed acrylic-based copolymer nanoparticles possess significant credentials concerning green technology as compared to those prepared with the conventional vinyl-based monomers in textile and garment sectors; however, their essential textile fastness properties are yet to achieve. In the present work, a series of acrylic nanodispersions were synthesized by varying the moles ratio of benzyl methacrylate (BZMA), methyl methacrylate (MMA), and 2-hydroxypropyl methacrylate (HPMA) monomers. This was done to study their effect on dye aggregation and dyed polymer particles agglomeration. FT-IR spectral analysis showed the formation of polymer structures, while Malvern Analyzer, Transmission Electron Microscopy, and Scanning Electron Microscopy analysis suggested that the particles are spherical in shape and their size is less than 200 nm. The obtained nanodispersions were later applied on cotton fabrics for the evaluation of wash fastness and colour migration. Premier color scan spectrophotometer and zeta potential measurement studies suggested that colour migration of printed cotton fabrics increased with an increasing agglomeration of particles and it was also observed to increase with the moles ratio of MMA and zeta potentials.

## 1. Introduction

Daylight fluorescent polymeric dispersions absorb radiation in the ultraviolet and visible regions of the electromagnetic spectrum and emit light in the visible region along with normal reflected light [1]. This makes them look brighter and hence gives them a glowing appearance. By carrying out the miniemulsion and microemulsion polymerization techniques in the presence of fluorescent organic dyes and ethylenically unsaturated aliphatic/aromatic monomers, one can form fluorescent dispersions containing dyed polymeric nanoparticles [2]. These materials have many applications and a huge market in many fields. For example: printing on textiles, the printing of advertising offers on bill-boards along highways, printing text and images in magazines, fluorescent signboards, fluorescent packaging at supermarkets for promoting consumer goods, for safety inscriptions on trucks, ambulances, fire trucks, and rescue equipment to mark boundary areas at dangerous places, traffic cones, worker safety vests, security inks, injection moulded toys, offset- printing, gravure, and flexo printing, industrial paints and many more. Among all these, developing materials for textile printing application is the most complicated task because the materials have to meet several requirements in order to be used for printing on textiles [3,4], Textile printing is a crucial process in the fabric industry to enhance the quality of the clothing and added value. Globally, printed textiles account for about 30–40% of all textiles [5]. The essential requirements are wash resistance to various washing detergents and wet/dry rub resistance [6,7,8]. Besides, various external mechanical forces including rubbing, agitation etc. and other environmental factors such as light, heat, chemicals strongly influenced the printed mateirals, which result in color change and undesirable color fastness [9].

To overcome this, several methods have been adopted including the development of disperse dye washing-free printing technology, which exploit the film-forming properties of polymer binder (PB) and the sublimation dyeing of disperse dye (DD). In this regard, Wang et al. have developed a novel washing-free printing binder through organosilicon modification of polyacrylate (PA) by mini-emulsion polymerization to achieve high color yield and color fastness of the printed fabric [10]. Typically, polyester fabric dyeing with DD occurred through the formation of “solid solution”, in which DD diffuses by a single molecule and dissolves into the pores of the amorphous region of polyester fiber [11]. Therefore, the dissolution behavior of DD into polyester has a strong influence on the solubility parameters of both DD and PB. For instance, in a recent study, a variety of polyacrylate (PA) binders were prepared by mini-emulsion polymerization using ethylhexyl acrylate (EHA) and methyl methacrylate (MMA) as monomers, which were selected based on the solubility parameter theory [8]. Results revealed that the larger solubility parameter differences between monomers and DD improved the color yield of the washing-free printed polyester fabric to a considerable extent.

Typical examples for ethylenically unsaturated monomers used in miniemulsion and microemulsion polymerization include vinyl compounds (styrene, α-methylstyrene, halogenated styrene (St), vinyl chloride, acrylonitrile (ACN), methacrolein, etc.) and acrylate esters (BZMA, MMA, 2-hydroxyethyl methacrylate, HPMA, butyl acrylate, methyl methacrylic acid allyl methacrylate, ethyleneglycol dimethacrylate, trimethylolpropane trimethacrylate, 2-hydroxy-3-chloropropyl methacrylate, glycidyl methacrylate etc.). The fluorescent dyes are typically triphenylmethane, indigo id, perylene, anthraquinone, rhodamine based dyes (Basic Violet 10, Basic Red 1:1 (BR), Basic Violet 11:1 (BV), rhodamine B, etc.), basic dyes such as Basic Yellow 2, Basic Yellow 40, Basic Green 1, Basic Green 4, etc. and solvent-based dyes (Solvent Yellow 14, Solvent Yellow 33, Solvent Yellow 82, Solvent Yellow 93, Solvent Yellow 124, Solvent Yellow 135, Solvent Yellow 160, etc.) [12,13,14,15].

The physicochemical properties of these fluorescent dispersions depend upon the degree of self-aggregation (which may be in the form of dye crystals or aggregates), the degree of molecular encapsulation of dye molecules, structure, and geometrical form of the polymer (sphere, worm or vesicle). In addition to this, the other parameters that they depend upon are particle size and distribution, and agglomeration of dyed particles. During the preparation of the dyed polymers, the most important step is to control the agglomeration of the dye molecules, which is the major reason for lowering the fastness properties of the printed fabrics. Molecular encapsulation of the fluorescent dyes with the polymer is essential for better fastness properties. Besides this, the fastness properties also depend on the selection of unsaturated monomers, their moles ratio, their interaction with dyes, reaction conditions, degree of encapsulation, structure and geometrical form of the polymer (sphere, worm or vesicle), particle size, and distribution, etc. [16,17,18,19]. The high moles ratio of hydrophobic monomer (less polar) results in poor encapsulation of the fluorescent dye molecules, while the high moles ratio of hydrophilic (polar) unsaturated monomers enriches the encapsulation of fluorescent dye molecules because of polar interactions between the fluorescent dye molecules and the polymer particles. In addition to the fluorescent dyes and polymer, other components for microemulsion polymerization are emulsifying agents, emulsion stabilizers, which induce electrostatic interactions and hydrogen bonding among the fluorescent dye molecules and the resulting polymer [20].

As the conventional monomers, St and ACN have environmental concerns, in the present work, we have used relatively environmentally friendly monomers, such as BZMA, MMA, and HPMA, while a combination of BR and BV were used as fluorescent dyes. The selected polymerizable monomer units produce fluorescent dispersions, which are much brighter and efficient in emission color as compared to the conventional fluorescent dispersions produced generally by using St, ACN, 2-hydroxyethyl methacrylate. The structural composition of the specified monomers regulates the morphological encapsulation of the fluorescent dyes and thereby improves the fluorescence emission color of the printed fabrics.

**Figure 1:**
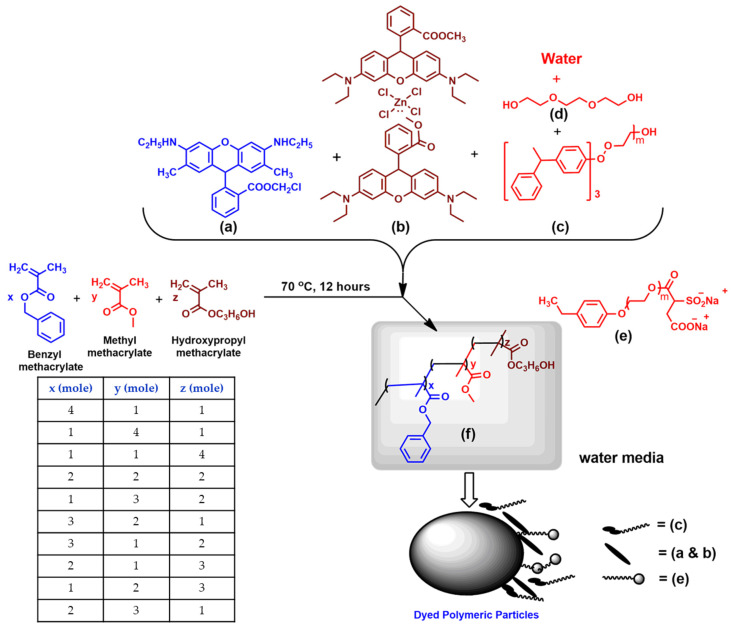
Graphical representation of a series of acrylic nanodispersions were synthesized by varying the moles ratio of BZMA, MMA, and HPMA monomers. (**a**) Basic Red 1:1, (**b**) Basic Violet 11:1, (**c**) Styrenated phenol ethoxylate, (**d**) triethyleneglycol, (**e**) A103 surfactant, and (**f**) polymeric particle, and the schematic of dyed polymeric particle.

We have developed a series of fluorescent polymer nanodispersions using free radical microemulsion polymerization. The resulting dispersions were found to have spherical-shaped particles. The dispersions were characterized by using solid content measurements, particle size analysis, UV-Visible, etc. The resulting dyed dispersion was applied on textile fabric and their wash fastness and color migration properties were evaluated. The representative structures of the synthesized polymers are shown in Figure 1 and the moles ratio of the monomers employed in different cases is provided in Table 1. In all cases, the free radical microemulsion polymerization continued in the presence of fluorescent dyes and the resultant formed dyed polymer particles are well stabilized in the water phase electrostatically by using an ionic surfactant, A103, and sterically by using a nonionic surfactant, styrenated phenol ethoxylate, 20 moles (SP20). The polymerizable unsaturated monomer units, BZMA, MMA, and HPMA are being used in the free radical microemulsion polymerization forming different structural compositions of polymers by differing the mole ratio of x, y, and z, as shown in Figure 1. In Figure 1, x, y, and z represent the moles of the corresponding monomers used in the free radical microemulsion polymerization, and accordingly, different copolymers were formed by changing their mole’s compositions, having the general typical structure represented in Figure 1.

## 2. Results and Discussions

### 2.1. UV-Vis Absorption of Rhodamine Dyes

**Figure 2:**
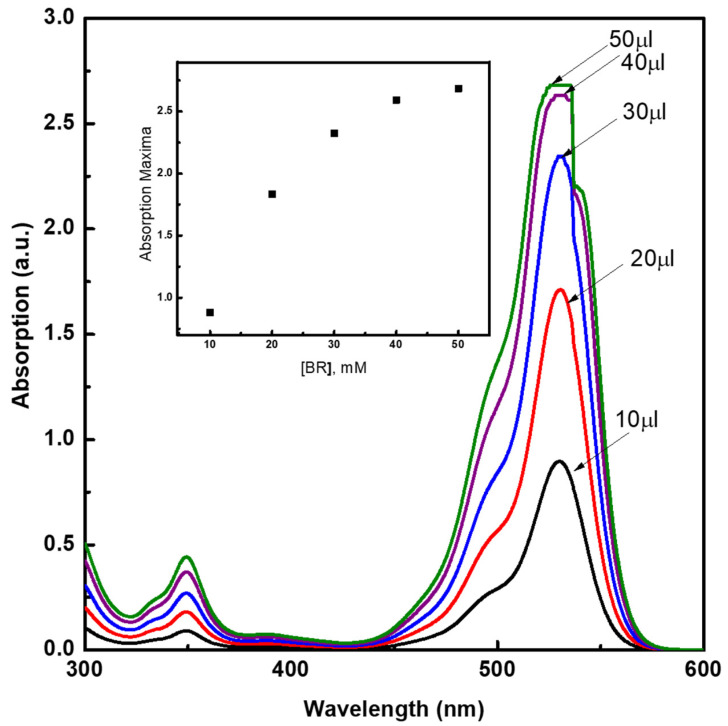
Absorption spectra as a function of the concentration of BR in the monomer mixture, prepared with 1:3:2 moles ratio of BZMA, MMA, and HPMA.

**Figure 3:**
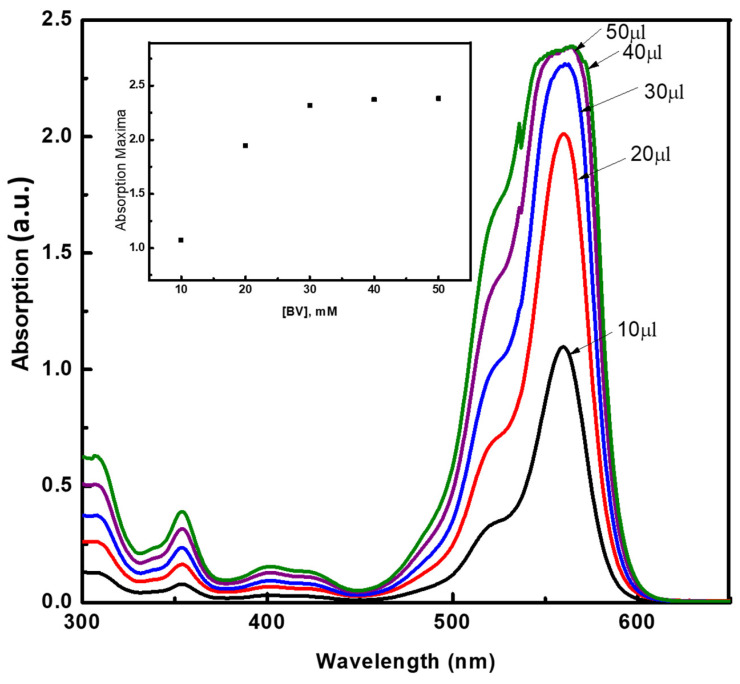
Absorption spectra as a function of the concentration of BV in the monomer mixture, prepared with 1:3:2 moles ratio of BZMA, MMA, and HPMA.

In order to study the self-aggregation phenomena of BR and BV in BZMA, MMA, and HPMA, absorption measurements were conducted and plotted against the moles ratio of each monomer. Stock solutions having concentrations in the range of 0.2→3.13 mM of BV and BR were prepared separately in DI water. Prior to absorption measurements, glacial acetic acid (0.01 weight percent to monomers) was used to increase the solubility of the dyes. The absorption maxima were recorded with increasing the volume of the stock solution from 10 to 50 µL and the concentrations of the resultant solutions range in between 5.8 to 29 µM. Figure 2 shows a relatively increased shoulder absorption band maxima at 498 nm, which corresponds to the dimer, while 524 nm corresponds to the monomer of BR [5,6]. The absorption spectra recorded as a function of the concentration of BV (Figure 3) showed a relatively increased shoulder absorption band maxima at 498 nm, which corresponds to the dimer, while 524 nm corresponds to the monomer of BV [21,22]. The absorption maxima values were found to increase with the increasing concentration of BV and BR. As the concentration reached around 15 µM, a deviation from Beer’s law was observed due to strong self-aggregation of BV and BR molecules, which are rich in π-conjugation [16,22,23,24,25,26,27,28,29,30].

### 2.2. Synthesis of Fluorescent Dispersions

Fluorescent dispersions were synthesized in accordance with the procedure given in the Experimental Section [18,31]. Reaction parameters such as moles ratio, type, and concentration of initiator, emulsifier, emulsion stabilizer, reaction temperature, etc. were initially studied to obtain the necessary conditions to be satisfied by fluorescent nanodispersions. The other reaction parameter is mechanical agitation, which has a significant influence on the agglomeration of particles during their formation. In the current method, fluorescent dispersions were synthesized with continuous feeding of monomers and initiator under low mechanical agitation, to avoid the formation of aggregate and coagulum, especially when the percent solids increase beyond 23% [32,33,34]. The stabilization of particles formed during microemulsion polymerization is of crucial importance as it severely leads to coagulation. All fluorescent dispersions were synthesized under identical optimized conditions except for the variation in the moles ratio of BZMA, MMA, and HPMA [19,24,28,32]. The ratio of ionic and non-ionic surfactants were optimized to increase the stability of the particles.

**Figure 4:**
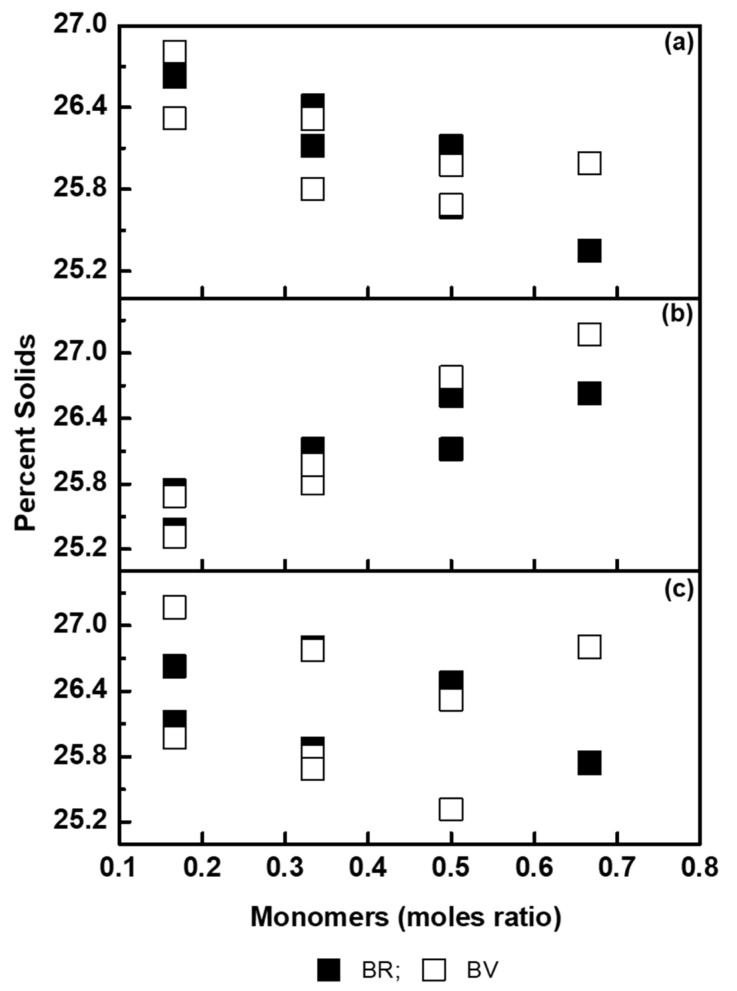
Percent solids of fluorescent dispersions formed during microemulsion polymerization as a function of moles ratio of (**a**) BZMA, (**b**) MMA and (**c**) HPMA.

Prior to rotary evaporation, the coagulum formed during microemulsion polymerization and the percent solids of the resultant fluorescent dispersions were plotted against the ratio of BZMA, MMA, and HPMA (Figure 4). The moles ratio was found to be influencing the percent solids. An increasing moles ratio of BZMA was found to decrease the coagulum formed, and this could be due to an increase in the solubility of higher aggregates of BV and BR. The increasing moles ratio of MMA showed an increase in the coagulum and percent solids, indicating relatively higher reactivity. During polymerization, the reactivity of acrylic ester monomers depends on the size and type of the alkyl group and is likely to decrease with the increasing size of the alkyl group. The methyl group in MMA makes it relatively more reactive leading to the formation of a coagulum. The higher moles ratio of MMA may increase the average molecular weight of the polymer formed [35], and this may lead to better encapsulation of rhodamine dyes with the resultant polymer. This may increase the dissolution of higher aggregates, thereby leading to a better encapsulation of the dye molecules. In the case of HPMA, percent solids were found to decrease with increasing moles ratio, which could most likely be favouring the control of the average molecular weight [36]. In the case of absorption measurements, increasing the moles ratio of HPMA decreases the absorption maxima.

### 2.3. FT-IR Analysis of Fluorescent Dispersions

**Figure 5:**
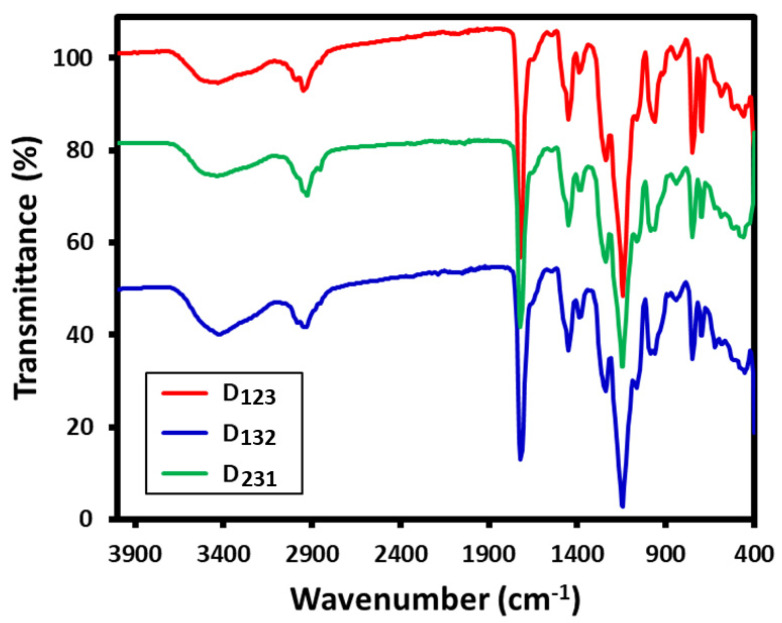
FT-IR spectra of D_123_, D_132_, and D_231_ synthesized without BR and BV.

Dispersions were synthesized without BV and BR in accordance with the procedure described in the Experimental Section and the resultant water-based dispersions were dried under high vacuum to obtain fine polymeric powders. As the structure of the resultant copolymers was characterized and confirmed by FT-IR analysis [31,37], the present resultant polymeric powders were characterized by FT-IR, and the corresponding resultant spectra are shown in Figure 5. The stretching bands at around 1722 cm^−1^ indicate the presence of C=O groups while stretching bands at 1028 cm^−1^ and 1452 cm^−1^ indicate the presence of C=C aryl rings and the CH-stretching band at 1360 cm^−1^ indicates the presence of aryl units. Stretching bands at 2880 cm^−1^ indicate the presence of aliphatic C-H groups. The broad stretching band at around 3200 cm^−1^ to 3600 cm^−1^ is the O-H group and H-bonding formation, respectively. These spectral results indicate the formation of copolymers with BZMA, MMA, and HPMA.

### 2.4. UV-Vis Absorption of Fluorescent Dispersions

**Figure 6:**
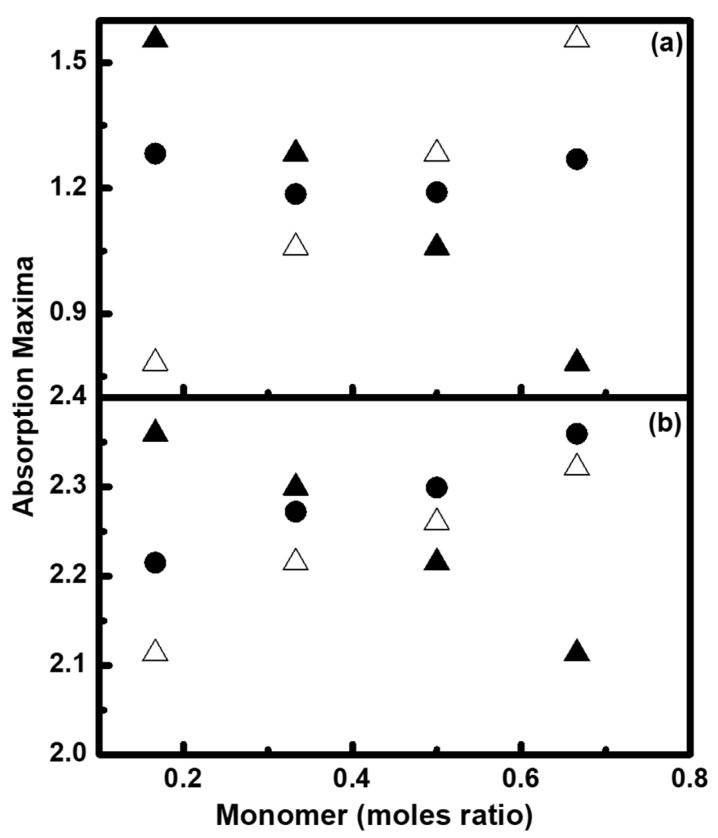
Absorption maxima of fluorescent dispersions as a function of moles ratio of BZMA (Δ), MMA (▲), and HPMA (•) for (**a**) BR and (**b**) BV.

The newly synthesized fluorescent dispersions were diluted 1000 times with DI water prior to absorption measurements. The absorption maxima were found to shift from 232 nm to around 350 nm for BV and 420 nm to around 540 nm for BR. Encapsulation of BR and BV with resultants is expected to shift the absorption maxima towards longer wavelengths. The absorption maxima plotted as a function of the moles ratio of BZMA, MMA, and HPMA are shown in Figure 6. As expected, a relative increase in absorption maxima for BV was found with an increasing moles ratio of BZMA. Fluorescent dispersions synthesized with BR do not show much effect on the magnitude of absorption maxima, but shift the absorption maxima towards a longer wavelength.

The formation of self-aggregation of dye molecules during microemulsion free radical polymerization and the formation of agglomerates of resultant polymeric particles during their synthesis influence the coloristic property of printed textile fabrics. The absorption maxima for both BR and BV were found to increase with the increasing moles ratio of BZMA. Hydroxypropyl/ethyl methacrylates, hydroxyl or polyhydroxyalkyl methacrylates with an increased moles ratio are more prone to form hydrogel type structures and have a tendency to swell by increasing the volume more than seven times, especially when treated with surfactant solutions or basic solutions.

### 2.5. SEM and TEM Analysis

**Figure 7:**
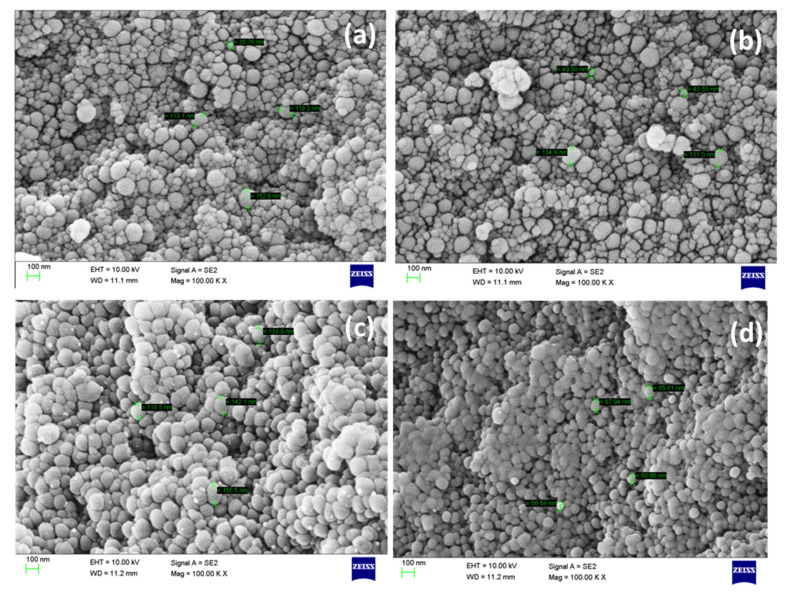
SEM images of (**a**) BRD_132_, (**b**) BRD_141_, (**c**) BVD_132_, and (**d**) BVD_141_ fluorescent dispersions.

In order to study the influence of moles ratio of BZMA, MMA, and HPMA on the morphology of polymer particles and to confirm the particle size, SEM and TEM analysis were conducted. Prior to analysis, BRD_132_, BVD_132_, BRD_141,_ and BVD_141_ fluorescent dispersions were allowed to pass through 200 nm pore size 3MM filter membranes and the resultant dispersions were kept in a preheated oven at 80 °C for 4 days and after this they were allowed to form fine powders by using the air sprayer technique. The preheating of these fluorescent dispersions confirms the stability of the nanodispersions. Figure 7a,c shows the SEM images of BRD_132_ and BVD_132_, while Figure 7b,d shows the SEM images of BRD_141_ and BVD_141_.

**Figure 8:**
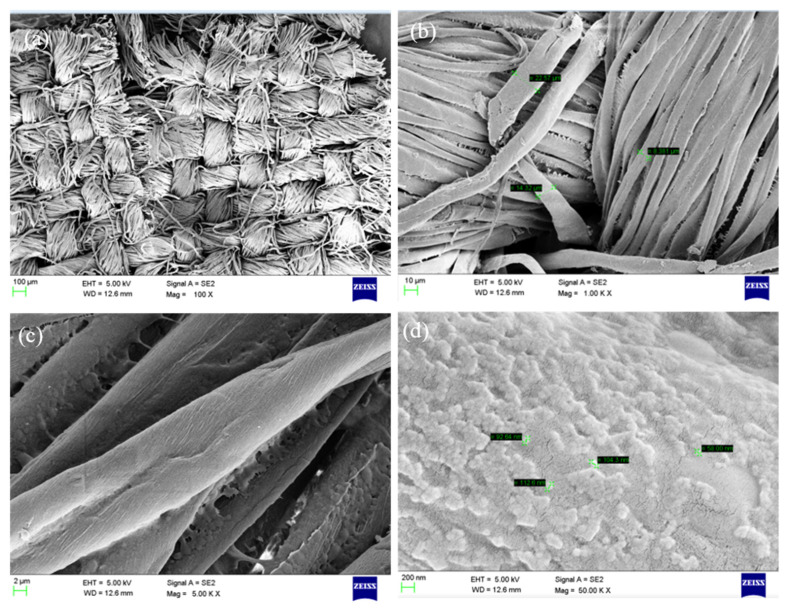
SEM images of (**a**) BVD_141_ printed cotton fabrics, (**b**,**c**) increased resolution, and (**d**) after curing at 150 °C per 5 min.

The spectroscopic studies show that the fluorescent dispersions produced by microemulsion free radical polymerization seem to be more appropriate for obtaining particles in nanosize with a narrow distribution [38]. As the liquid dispersions prior to SEM analysis were converted into a powders form, it is unable to see the agglomeration of the particles in SEM analysis. However, as concerns the geometrical structure, the dyed polymer particles are homogeneous and spherical in shape. The morphology of polymer particles hardly shows any change with respect to BV and BR. The size of the particles ranges from 35 to 155 nm. This clearly indicates that the particles are below 200 nm, which is obvious as they are passed through 200 nm 3MM filter membranes. The printed cotton fabrics of BVD_141_ were shown in Figure 8a–c while Figure 8d shows the cured cotton fabric at 150 °C per 5 min.

**Figure 9:**
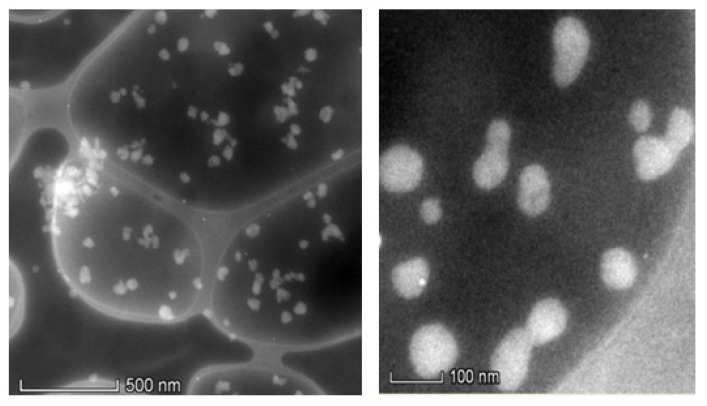
TEM images of colored BVD_141_ dyed polymeric particles after thermal treatment at different scale bars.

BVD_141_ fine powder was analyzed by TEM analysis and the resultant images were shown in Figure 9. As concerns the particle size, the TEM analysis further confirms the particles size and also the spherical shape of the particles. The close observation of the images shows little agglomeration of particles, which obviously did not affect the filtration of the fluorescent dispersion through the 200 nm 3MM filter membrane. This indicates that the agglomeration, which is present to a small extent, is not rigid nor found to affect the structural properties.

### 2.6. Particle Size Distribution Curves

The size and distribution of particles of fluorescent dispersions determine the quality of printed textile cotton fabrics. The tinctorial strength of the printed cotton fabrics, especially the color depth, increases by 20–27% relatively by reducing the particle size below the submicron levels. The molecular weight and concentration of emulsion stabilizer, type and concentration of monomers and initiators, polymerization temperature, ionic strength, type and concentration of fluorescent dyes encapsulated in particulate form, etc. influence the size of particles and their distribution [39,40]. In addition to this, the agglomeration phenomenon of dyed polymer particles either during the microemulsion free radical polymerization or during the continuous mechanical agitation or during long-term storing affects chemo-physical properties and also the performance of printed textile cotton fabrics. One of the reasons for particles agglomeration could most likely be the tendency of aggregation of fluorescent dye molecules during microemulsion polymerization [41]. Hopefully, it could be possible to control, either by improving the interactions between the dye molecules and polymers or by increasing the crosslink density of the polymer (using bi-/tri- functional acrylic cross-link ester monomers, such as EGDMA, allylmethacrylate, trimethylolpropane trimethacrylate, etc.) [42,43].

**Figure 10:**
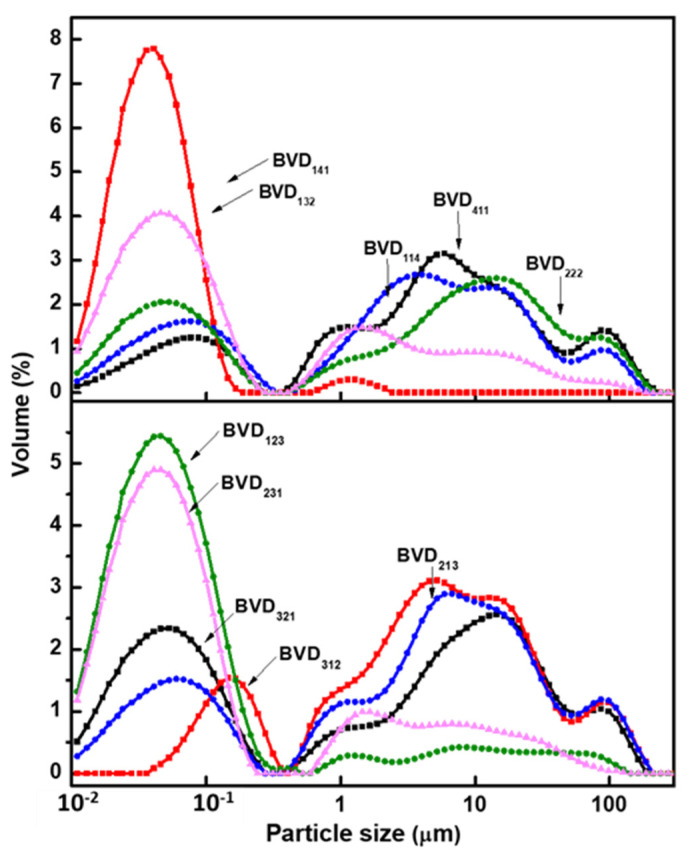
Particle size distribution curves of fluorescent dispersions synthesized with BV.

**Figure 11:**
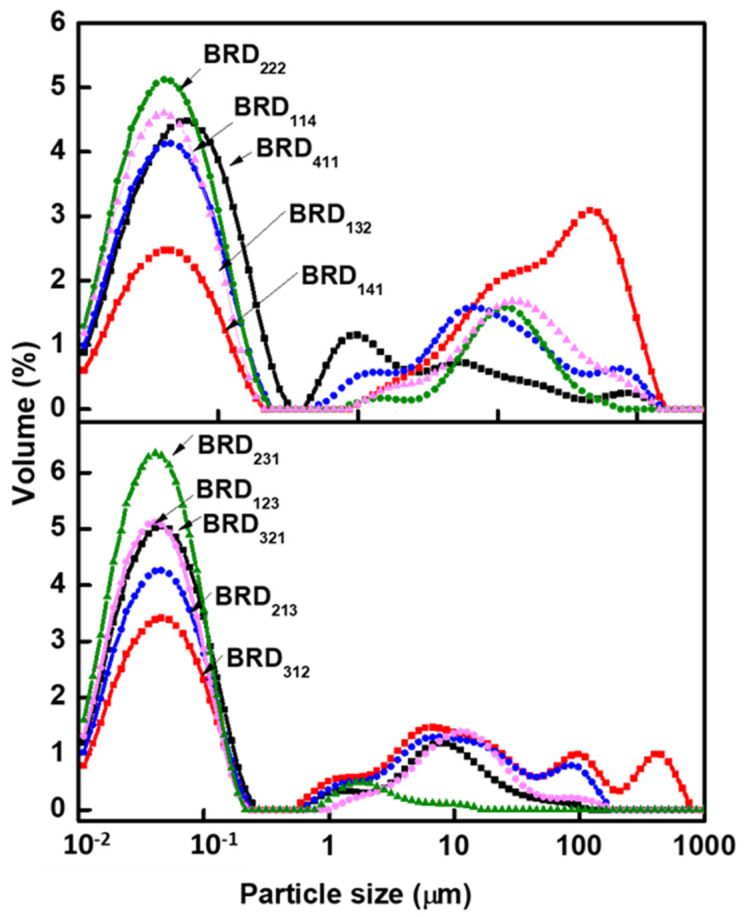
Particle size distribution curves of fluorescent dispersions synthesized with BR.

As the ultrasonic technique along with high dilution (total solid content during the free radical polymerization is below 18%) was used in the present work, the resultant formed dyed polymeric particles were assumed to be in the nano range. After completion of filtration, the resultant dispersions were analyzed by the Malvern analyzer and the corresponding curves, ranging from 0.01 to 100 µm, as shown in Figure 10 and Figure 11. This indicates that the formed nanoparticles undergo agglomeration and the resultant curves in Figure 10 and Figure 11 correspond to agglomerated particles. The Dv (90) of agglomerated particles for all the dispersions is in the range 62–80 µm, which means 90% of the agglomerated particles have a size less than this value. In addition, the major observation from this analysis is that with the increase of hydrophilic polar monomer moles ratio in the polymer, the agglomerated particle size of the polymer particle decreases. For example, by comparing the particle size of the basic violet dye-based dispersions such as BVD_114_, BVD_141_, and BVD_411_ provide information about the effect of particular monomer (such as BZMA, MMA, and HPMA) on the size of agglomeration. Among these three, BVD_411_ and BVD_141_ dispersions exhibited sizes of agglomerated particles, D_v_(90) ranging from 60 to 72 µm, while BVD_114_ dispersions exhibited D_v_(90) ~35 µm. The first two dispersions BVD_411_ and BVD_141_ have high moles ratio of hydrophobic BZMA and MMA monomers, whereas the third dispersion BVD_114_ shows less and controlled agglomerated particles due to the use of a high moles ratio of hydrophilic monomer HPMA.

As compared to BVD_141_, the dispersions prepared with the decreased moles ratio of MMA and the increased moles ratio of HPMA showed good control on the size of agglomerated particles, when D_v_ (90) was reduced from 35 to ~11 µm. As compared to BVD_411_, BVD_321_ showed slightly reduced agglomeration. The BVD_411_ exhibited D_v_ (90) from 60 to 42 µm in the case of BVD_321_. Therefore, the reduction of agglomeration could be brought about either by decreasing the moles ratio of BZMA or MMA.

From the earlier examples, it is seen that increasing the moles ratio of HPMA or decreasing the moles ratio of MMA favored the control of the agglomeration of particles. However, BVD_312_ and BVD_321_ showed similar curve patterns of agglomeration. This could be because at a higher moles ratio of BZMA, the influence of MMA and HPMA on agglomeration will be minimized as compared to the effect of BZMA. By reducing the moles ratio of BZMA from 4→3→2, the fluorescent dispersions (BVD_213_ and BVD_222_) showed a high agglomeration of particles.

The dyed polymeric particles were found prone to agglomeration with an increasing moles ratio of BZMA. This could most likely be due to the hydrophobicity of BZMA. The polarity of the reaction medium influences the agglomeration of particles, which decreases with increasing polarity [44,45]. A gradual increase of the moles ratio of BZMA is expected to result in a reduction of microemulsion mean droplet size and thus leads to the formation of high moles ratio of hydrophobic seed particles, which are more unstable, hence leading to agglomeration [39,46].

A controlled agglomeration of particles was seen in the case of fluorescent dispersions synthesized with the high moles ratio of HPMA. As it is known, HPMA is an amphiphilic and self-emulsifying agent and also effectively stabilizes the resultant fluorescent emulsions. Hydroxyl groups of HPMA are prone to form H-bonds and its higher moles ratio in a polymer may lead to flocculation or coagulum formation [47]. Optimum levels of HPMA may balance stabilization forces at the interface between water and the particles thereby, which may control the agglomeration. An increasing moles ratio of HPMA could be expected to emulsify the microemulsions better compared to those of BZMA and MMA. Increasing the average molecular weight and concentration of PVA increases the stability of microemulsion during the polymerization and also controls the agglomeration of particles [44]. The BVFD_123_ fluorescent dispersions have shown the best control of agglomeration. HPMA emulsifies the microemulsions efficiently and stabilizes the resulting particles through inter-molecular H-bonding and Vander Waals forces.

The particle size distribution curves of the fluorescent dispersions synthesized with BR are presented in Figure 11. When comparing the particle size distribution of BR relative to BV, (Figure 10 and Figure 11), it can be noted that the conditions favorable for better control of agglomeration of particles with BR are unfavorable for those particles of BV under identical conditions and vice versa. As in the case of fluorescent dispersions synthesized with a 1:4:1 moles ratio of BZMA, MMA, and HPMA, BV shows better control of agglomeration, while BR shows a higher level of agglomeration. As it is seen from Figure 10 and Figure 11, increasing the moles ratio of BZMA decreases the agglomeration of particles for BR, while it was observed to increase when increasing the moles ratio of MMA and HPMA.

**Figure 12:**
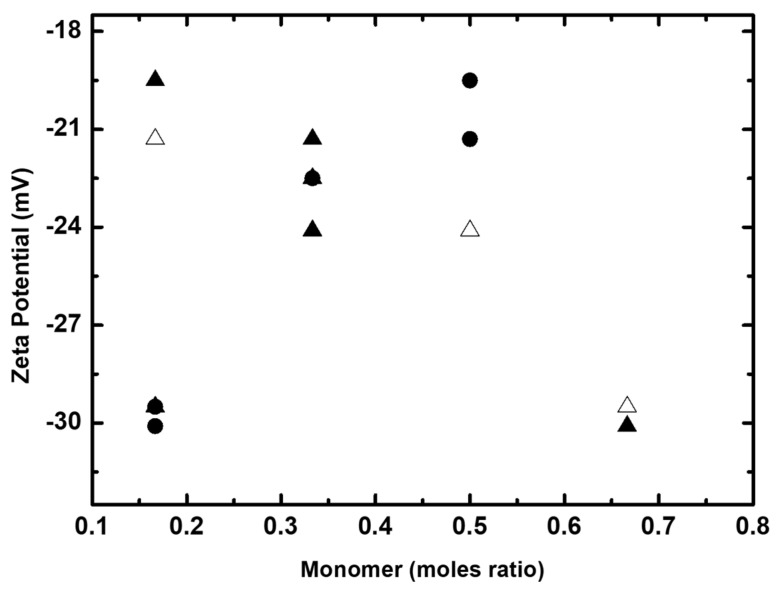
Zeta potential as function of moles ratio of BZMA (Δ), MMA (▲) and HPMA (•).

Malvern Zetasizer nano series was used to take the measurements of zeta potentials of various acrylic polymer dispersions, in order to know the stability, relative shelf life of the dispersions, and also the interactions of these acrylic polymer particles with the rhodamine dyes. The charge acquired by the particles depends on the type of monomers used and also on the rheological structural properties of the polymers. In the current study, the monomers selected are non-ionic and the resultant polymer particles exhibit surface polarity rather than surface charge. The charge acquired by these polymer particles are generally low when compared to those formed with partially substituted acrylic ionic monomers, whose zeta potentials increase with increasing pH [48]. With an increasing pH, the ionic groups ionize and generate ionic charges around the surface of the particles. In the current study, the monomers are non-ionic and the resultant particles possess surface polarity. The zeta potentials of these dispersions were expected in the moderate range of −20 to −30 mV. The stability of these dispersions could be increased sterically by increasing CH_3_ or -CH_2_-C_6_H_5_ groups. It is seen from Figure 12 that increasing the moles ratio of MMA and BZMA is found to increase the zeta potentials and this could be due to an increase in the stability of the particles by substitutions of the CH_3_ group in MMA or the CH_2_C_6_H_5_ group in BZMA. These substitutions balance surface hydrophobic interactions with less favorable hydration and thus stabilize the particles sterically. There is a decrease in the zeta potential of dispersions when prepared with increasing moles ratio of HPMA, due to the additional parameter of hydrogen bond formation among the particles.

### 2.7. Wash Fastness and Color Migration Properties

**Figure 13:**
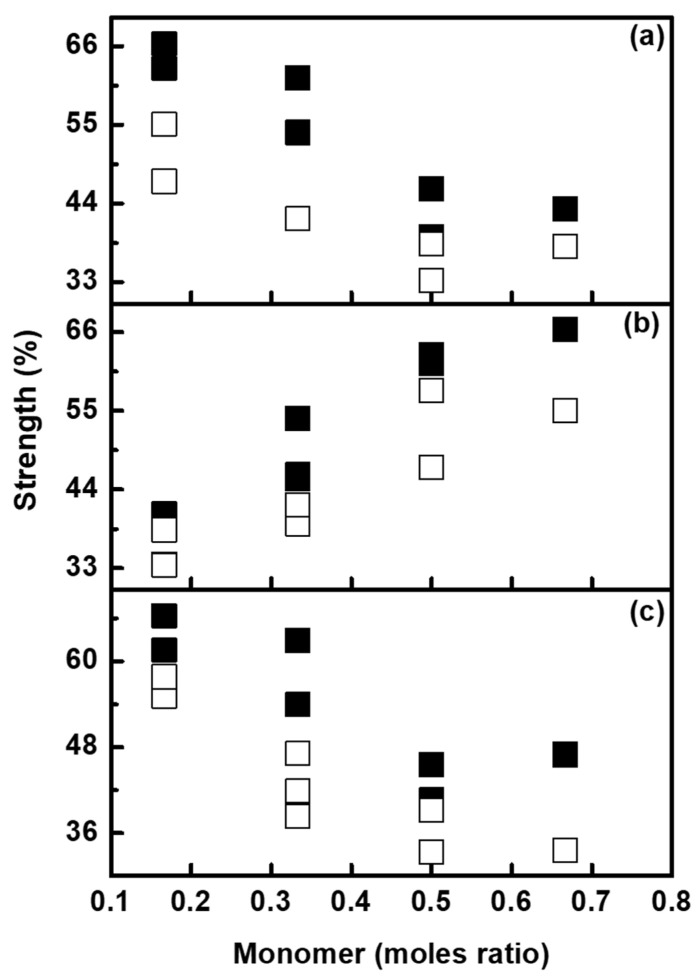
Strength of printed cotton fabrics after washing; BR (**□**) and BV (**■**), as a function of moles ratio of (**a**) BZMA, (**b**) MMA and (**c**) HPMA.

**Figure 14:**
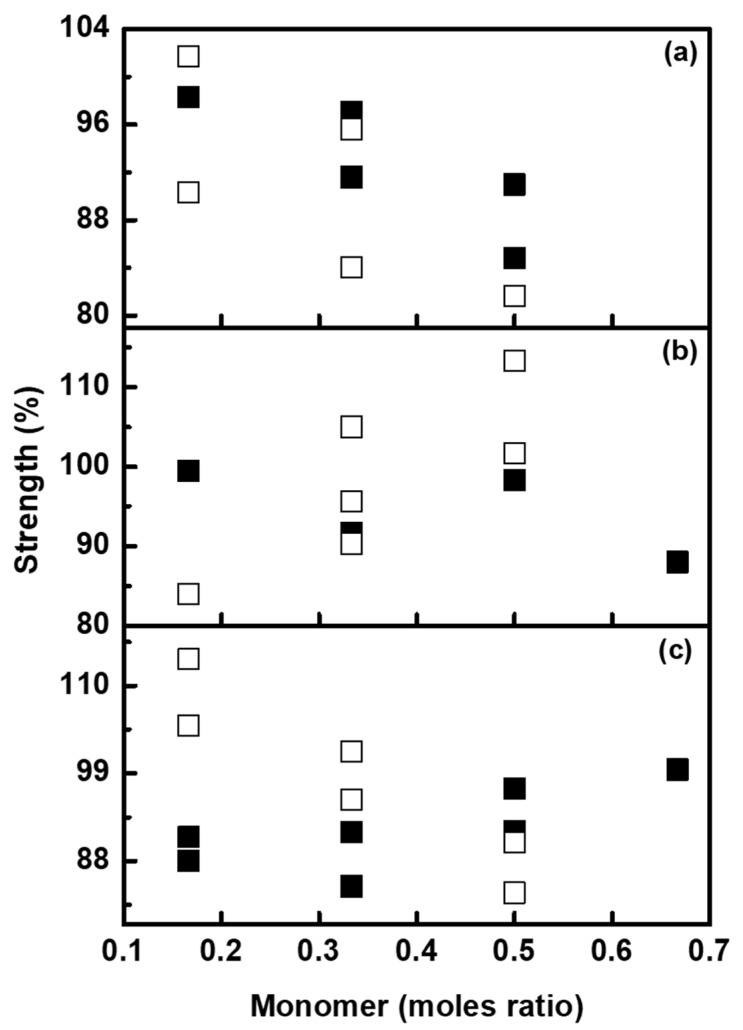
Strength of printed cotton fabrics after color migration; BR (□) and BV (■), as a function of moles ratio of (**a**) BZMA, (**b**) MMA and (**c**) HPMA.

After adjusting the total solid content to around 40%, the resultant fluorescent dispersions were used to prepare printing pastes as per the procedure given in the Experimental Section. In the textile application, the critical fastness properties are wash, Saliva, perspiration, and wet/dry. Measurements of color fastness to washing and color migration were conducted as per the procedures described in the Experimental Section. The critical property related to the last three Saliva, perspiration, and wet/dry properties, is color migration from the printed coloured fabric to the unprinted fabric either through water media or by rubbing the coloured fabric surfaces. Measurements taken by water media have values of low uncertainty as compared to those of wet/rub and accordingly, measurements of color migration were conducted as per the procedure described in the Experimental Section. Figure 13 and Figure 14 shows the strengths of the washed fabrics and corresponding color migration of the fabric samples, printed with the fluorescent dispersions. The images of BV and BR prints of BRD_132_, BRD_141_, BVD_132_, and BVD_141_ before and after washing and color migration into unprinted fabric are shown in Supplementary File (Figures S1 and S2).

Agglomeration of polymeric particles influences the dyeing ability of copolymers with the dye molecules and is expected to be poor in case of the high degree of agglomeration. The agglomerated particles will not allow the dye aggregates to dissolve molecularly, thereby encapsulation of such dye aggregates results in poor performance on printed cotton fabrics during their evaluation of fastness properties, especially washing and colour migration, and thereby play an important role in determining the quality of printed cotton fabrics [49].

Wash fastness is defined as the degree of resistance of migration of color from printed cotton fabric to non-printed cotton fabric during thermal washing or hydrothermal treatment. Several parameters, especially the type and concentration of surfactants used, temperature, time, etc. influence the washing performance [50]. It was observed that, the tinctorial strengths were found to decrease with an increasing moles ratio of BZMA and HPMA, whereas they were found to increase with an increasing moles ratio of MMA (Figure 13). The change in the shade of the cotton fabrics which were printed with fluorescent dispersions synthesized with high moles ratio of BZMA and HPMA was higher than that of MMA. Increasing the moles ratio of MMA improves the overall washing performance, most likely due to better encapsulation of the dyes with the polymer and also by the use of binders synthesized with MMA [25]. Similarly, the colour migration strength was found to increase with the moles ratio of MMA and decrease with the moles ratio of BZMA and HPMA.

Another phenomenon expected to occur in fluorescent nanodispersions is the agglomeration of particles. During microemulsion free radical polymerization, the agglomeration of particles induces aggregation of rhodamine dye and thereby an effect on the encapsulation of dye molecules is expected. During washing, the aggregated dye molecules dissolve molecularly, causing color migration to non-printed cotton fabrics. To study the influence of the agglomeration of particles on color migration, the strengths of cotton fabrics obtained after washing were observed. As it is seen from Figure 14, the strengths were found to increase for both BV and BR dyes with the increasing agglomeration of particles.

**Figure 15:**
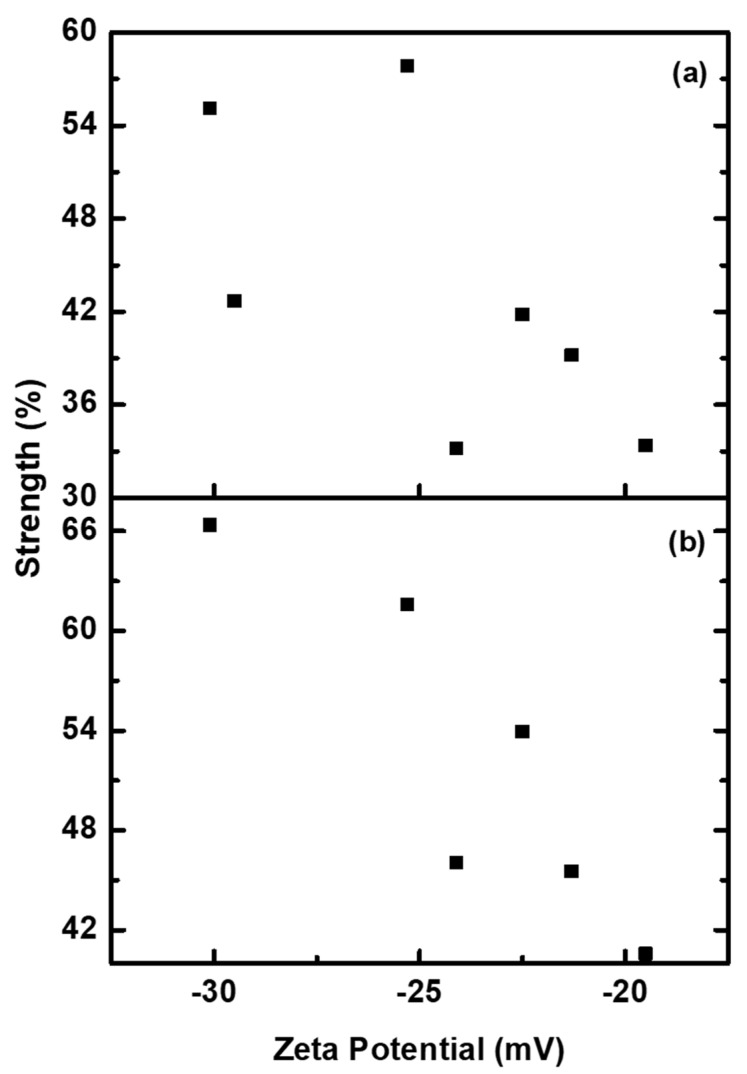
Strength of printed cotton fabrics after washing, (**a**) BR and (**b**) BV, as a function of zeta potential.

Figure 15 shows the zeta potentials of dispersions plotted against the strength (%) of washed BV and BR dispersion of printed cotton fabrics. It is seen from Figure 15 that the strength of the wash fastness of the printed cotton fabrics was found to increase with increasing zeta potentials of dispersions. This could most likely be due to an increase in the moles ratio of either BZMA or MMA, hence indicating the presence of a hydrophobic particle surface, which is less affected by the detergent solutions.

## 3. Materials and Methods

### 3.1. Chemicals and Materials

BZMA (96%), MMA (99%) and HPMA (99%), potassium per sulphate (KPS) (99%) (K_2_S_2_O_8_, Sigma-Aldrich, Bangalore, India), and sodium metabisulfite (SMBS) (97%) (Na_2_S_2_O_5_, Sigma-Aldrich), Acetic acid (99.8%) were used as received. Disodium salt of ethoxylated nonylphenol half ester of sulfosuccinic acid (Aerosol A-103) (34%) procured from Cytec, New Delhi, India) was used as received. Styrenated phenol ethoxylate (20 moles), procured from Venus Ethoxylate Pvt Ltd., Goa, India, was used as received. Sodium salt of 2-acrylamido-2-methyl-1-propanesulfonic acid solution (NaAMPS) (H_2_C=CHCONHC(CH_3_)_2_CH_2_SO_3_Na 50% (*Wt*/*Wt*.) in water, Sigma-Aldrich) was used as received. Poly (vinyl alcohol) (PVA) (number average molecular weight around 89,000–98,000, 99% hydrolyzed, Sigma-Aldrich) was used as an emulsion stabilizer. Deionized water with resistivity of ~18 MΩ cm was used as an inert medium for all the synthesis of fluorescent dispersions. PT 40, acrylic polymer thickener for dispersion printing, procured from Topaz Texchem Pvt. Ltd., Mumbai, India, was used as received. ET (polyacrylate) of BASF was used as a binder. All chemicals unless otherwise specified are of Laboratory Reagent and were used as received. Textile cotton woven fabrics (100%) having construction 23.6 ends/cm^2^ and an area density of 112.2 g/m^2^ was used as fabric for printing.

### 3.2. Synthesis of Fluorescent Dispersions

A 1000 mL five-neck round-bottom flask equipped with a reflux condenser, stainless steel stirrer, and two separate feed streams was used to carry out microemulsion free radical polymerization. Oxygen was removed by purging high purity nitrogen gas into DI water (500 g), which was transferred into the five-neck round-bottom flask. Then, the emulsifying agent, A-103 (10.0 g), SP20 (2.5 g) and PVA (0.2 g) were added and stirring was continued for about 30 min under a blanket of nitrogen at 80 °C. After this, the temperature was gradually allowed to reach room temperature. The aqueous solution of NaAMPS (6.6 g) was added. A premixture (12.0 g) previously prepared by mixing the corresponding quantities of BZMA, MMA, and HPMA was transferred to the round bottom flask. An aqueous initiator solution (7.5 g) prepared by dissolving KPS (1.3 g) and SMBS (0.08 g) in water (20.0 g) was charged into the reaction flask and stirred in the ultrasonic bath for 40 min. To the remaining portion of monomer mixture (feed monomer), the specified quantities of BR and BV were added and mixed under slow rpm. The resultant dye monomer solution was divided into 8 eqi-volume portions and each volume portion was added over a period of 60 minutes. After completion of addition of the monomer, dye solution, and the initiator solution, the polymerization was further continued at the same temperature for another 2 h. After this, tert-butyl hydroperoxide was added and the polymerization was further continued for another 2 h. The resultant dispersions were allowed to reach room temperature and then filtered through a stainless-steel fine mesh strainer sieve colander 7-5/8 inch to separate the coagulum formed during microemulsion polymerization. After completion of the filtration, the coagulum formed was separated and dried in the oven at 100 °C for 15 min and then weighed. The resultant dark magenta coloured fluorescent dispersion was subjected to rotary evaporation under vacuum (680 mmHg) to achieve percent solids ~40%.

### 3.3. Characterization and Measurements

#### 3.3.1. Measurement of Percent Solids

The resultant fluorescent dispersion (0.5 g) was weighed into a Borosilicate glass Petri dish (40 × 15 mm^2^) and then placed in a preheated oven at 120 °C for 30 min. After completion of 30 min, the dish was taken out and placed in a glass desiccator and allowed to reach room temperature. The percentage of solid was calculated after weighing the dish.

#### 3.3.2. Measurement of Absorbance

Absorption measurements were made by using Shimadzu UV-Vis Absorption Spectrophotometer, UV-1601PC model using 0.1 cm Quartz cuvettes. Prior to the absorption measurements, the fluorescent dispersions had been diluted to milli scale with DI water and had been subjected to ultrasonication to eliminate microbubbles.

#### 3.3.3. Measurement of Particle Size

The Malvern Mastersizer 3000 was used to measure particles of the newly synthesized fluorescent dispersions. Before taking the measurements, the fluorescent dispersions were subjected to ultrasonication to eliminate microbubbles. All scans were done at 1400 rpm of stirring. The medium values for the particle size distribution are presented in the form of D_v_(10), D_v_(50), and D_v_(90), where “D” usually stands for diameter distribution and “v” for a volume of either (10), (50), or (90), which represents the percentage of volume, below which the particular diameter is present. Malvern Zetasizer nano series was used to take zeta potential measurements of the dispersions.

#### 3.3.4. Observations by SEM and TEM Analysis

After filtration through the fine mesh strainer sieve colander, the dispersions were further filtered through Betafine DP Series Polypropylene Cartridge of pore size 0.2 µm, supplied by 3M Purification Inc, USA. Almost 99% of fluorescent dispersions could pass through it. The resultant dispersions were dried by using a sprayer gun and then dried at 100 °C for about 10 min. The SEM pictures were taken using the ESEM Quanta 200, FEI, W-Filament, with low vacuum and humidity capability, secondary E-T and solid state backscattered electron detector, and an ultra-thin window EDS system, with a resolution of 20 kv:3 nm in high vacuum. The TEM pictures were taken using JEOL 2000 FX-II with an ultra-thin window oxford instruments EDS system and a CCD image recording system.

#### 3.3.5. Printing Paste

The print paste was prepared by stirring DI water (85.5 g) and the binder (11.1 g) for 15 min at 180 rpm and after 15 min, liquid ammonia (0.5 g) was added and mixed slowly for another 15 min. To this, the thickener (0.3 g) was slowly added for 15 min at around 320 rpm of stirrer and stirring was continued further for 30 min.

#### 3.3.6. Printing Technique

The resultant paste (9.0 g) was mixed with the newly synthesized fluorescent dispersions (2.5 g of 40% solid) and then soaked for about 36 h. The homogenized printing paste was then applied to the cotton fabrics (previously cut into the specimen dimensions of 130 × 80 mm^2^) along the radial direction of fabric and baked at 80 °C for 5 min followed by curing at 150 °C for 3 min. The resultant printed cotton fabrics were cut into smaller pieces with a dimension of 110 × 60 mm^2^ and were then subjected to analysis.

#### 3.3.7. Measurement of Color Strength

The resultant specified cotton fabrics mentioned above were analyzed by Premier Color Scan Spectrophotometer, Model No. 5100. Prior to scanning, the instrument was calibrated, using a white tile for 100% reflectance.

#### 3.3.8. Measurement of Colorfastness to Wash

The printed cotton fabrics were washed using 2.0 weight percent “Surf excel” detergent solution prepared in DI water. The solution (20 times the weight of the printed cotton fabric) was rinsed at 60 °C for 10 min. After the fabric was rinsed twice with DI water (each time for 1 min with 25 mL DI water), it was squeezed and then dried in the oven at 100 °C. The dried cotton fabrics were scanned with Premier Color Scan Spectrophotometer to measure the variations in both strength (%) and shade (dE*).

#### 3.3.9. Measurement of Color Migration

Surf excel detergent liquid solution (2.0 weight percent) prepared in water was used for the measurement of color migration. After cutting the printed cotton fabrics to the specimen dimensions, the unprinted cotton fabrics of 60 × 25 mm^2^ were cut and both placed in a conical flask and the detergent solution (20 times the weight of the printed cotton fabric) was transferred and then rinsed at 60 °C for 10 min. Then, after the two cotton fabrics were rinsed twice with DI water (each time for 1 min with 25 mL DI water), they were squeezed and dried in the oven at 100 °C for 5 min. The dried cotton fabrics of 60 × 25 mm^2^ were scanned with Premier Color Scan Spectrophotometer to measure the variations in both strength and shade (dE*).

## 4. Conclusions

In the current investigation, the microemulsion free radical polymerization technique was employed to synthesize high-performance fluorescent dispersions (having nanosize BR/BV dyed polymer particles), which are free from the conventional vinyl monomers, such as styrene and acrylonitrile, which have an environmental concern. Moreover, this report also provides a correlation between the new environmentally friendly monomer’s mole ratio and the fastness properties of the printed cotton fabrics. Malvern analysis, SEM, and TEM analysis show the presence of homogeneous and spherically shaped particles having a size below 200 nm. The moles ratio of various acrylic monomers influences the aggregation of dye molecules, agglomeration of dyed polymeric particles, and ultimately influences the fastness properties of printed textile fabrics. The zeta potentials measured for these fluorescent dispersions are as moderate as the monomers used in the fluorescent dispersions are from non polar to mild polar. Dispersions synthesized with 1:4:1 and 1:2:3 moles ratio of BZMA, MMA, and HPMA respectively showed controlled agglomeration for BV, whereas for controlled agglomeration of BR, a 2:3:1 moles ratio was used. Increasing the moles ratio of HPMA was found to decrease the zeta potentials. Increasing the moles ratio of MMA was found to increase the zeta potentials of fluorescent dispersions and correspondingly the wash fastness properties. Further colour migration analysis showed its linear dependency on the volume percentage of agglomerated particles. In both BV and BR fluorescent dispersions, the volume percentage of agglomerated particles was found to vary linearly with color migration.

## Figures and Tables

**Table 1:** Experimental conditions and absorption maxima of resultant fluorescent dispersions.

BZMA (g)	MMA (g)	HPMA (g)	FD	BR		FD	BV	
				λ_max_ (nm)	Abs. Max.		λ_max_ (nm)	Abs. Max.
64.08	9.10	13.11	BRD_411_	532.5	1.554	BVD_411_	564.0	2.321
16.00	36.41	13.11	BRD_141_	531.9	0.782	BVD_141_	561.9	2.113
16.00	9.10	52.42	BRD_114_	532.4	1.269	BVD_114_	564.0	2.359
32.00	18.20	26.21	BRD_222_	531.9	1.280	BVD_222_	563.1	2.272
16.00	27.31	26.21	BRD_132_	532.0	1.186	BVD_132_	562.5	2.255
48.00	18.20	13.11	BRD_321_	532.0	1.282	BVD_321_	563.9	2.260
48.00	9.10	26.21	BRD_312_	531.9	1.193	BVD_312_	564.4	2.446
32.00	9.10	39.32	BRD_213_	532.4	1.129	BVD_213_	563.9	2.260
16.00	18.20	39.32	BRD_123_	532.0	1.190	BVD_123_	563.0	2.299
32.08	27.30	13.10	BRD_231_	532.0	1.058	BVD_231_	562.0	2.215

‘FD’ represents fluorescent dispersions, ‘D’ represents dispersion where the suffix numbers represent the moles ratio of the monomer units of BZMA, MMA, and HPMA respectively. The weights given in ‘g’ are proportional to the corresponding moles ratio given in the Table, Figure 1.

## Data Availability

The data presented in this study are available within the article and supplementary material.

## Supplementary Materials

The following are available online, Figure S1: (a) printed fabrics of fluorescent dispersions of BV132 and 141 (b) corresponding wash fastness (c) corresponding colour migration of printed fabrics to unprinted fabrics. Figure S2: (a) printed fabrics of fluorescent dispersions of BRD132 and 141 (b) corresponding wash fastness (c) corresponding colour migration of printed fabrics to unprinted fabrics. Figure S3: Schematic representation of a series of acrylic nanodispersions were synthesized by varying the moles ratio of benzyl methacrylate (BZMA), methyl methacrylate (MMA), and 2-hydroxypropyl methacrylate (HPMA) monomers. (a) Basic Red 1:1, (b) Basic Violet 11:1, (c) Styrenated phenol ethoxylate, and (d) A103 surfactant.

## Sample Availability

Samples of the compounds are available from the authors.
