# Supplementary material for: Synthesis of High-Performance Aqueous Fluorescent Nanodispersions for Textile Printing—A Study of Influence of Moles Ratio on Fastness Properties
Source: Molecules. 2021 Nov 23;26(23):7075. doi: 10.3390/molecules26237075 (PMC8658936; doi:10.3390/molecules26237075)
Supplement: Supplementary file 1 [file molecules-26-07075-s001.zip › molecules-1464502-supplementary.pdf]

molecules

Supplementary File

Synthesis of High-Performance Aqueous Fluorescent Nanodispersions for Textile Printing—A Study of Influence of Moles Ratio on Fastness Properties

Shruthi Manjunath Shenava [1,2,*], J. V. Shanmukha Kumar [2], Rajkumar Ganugula [1,*], Mohammed Rafi Shaik [3,*], Rosa Busquets [4] and Mohammad Rizwan Khan [3]

[1] Aron Universal Limited, #25/1, 2nd phase, Jigani Industrial Area, Jigani, Bangalore 560105, Karnataka, India
[2] Department of Chemistry, Koneru Lakshmaiah Education Foundation, Vaddeswaram, Guntur 522502, A.P., India; shanmukh_fed@kluniversity.in
[3] Department of Chemistry, College of Science, King Saud University, P.O. Box 2455, Riyadh 11451, Saudi Arabia; mrkhan@ksu.edu.sa
[4] School of Life Sciences, Pharmacy and Chemistry, Kingston University London, Penrhyn Road, Kingston-upon-Thames, Surrey KT1 2EE, UK; r.busquets@kingston.ac.uk
* Correspondence: shruthimshenava@gmail.com (S.M.S.); raz29in@gmail.com (R.G.); mrshaik@ksu.edu.sa (M.R.S.); Tel.: +966-11-46-70439 (M.R.S.)

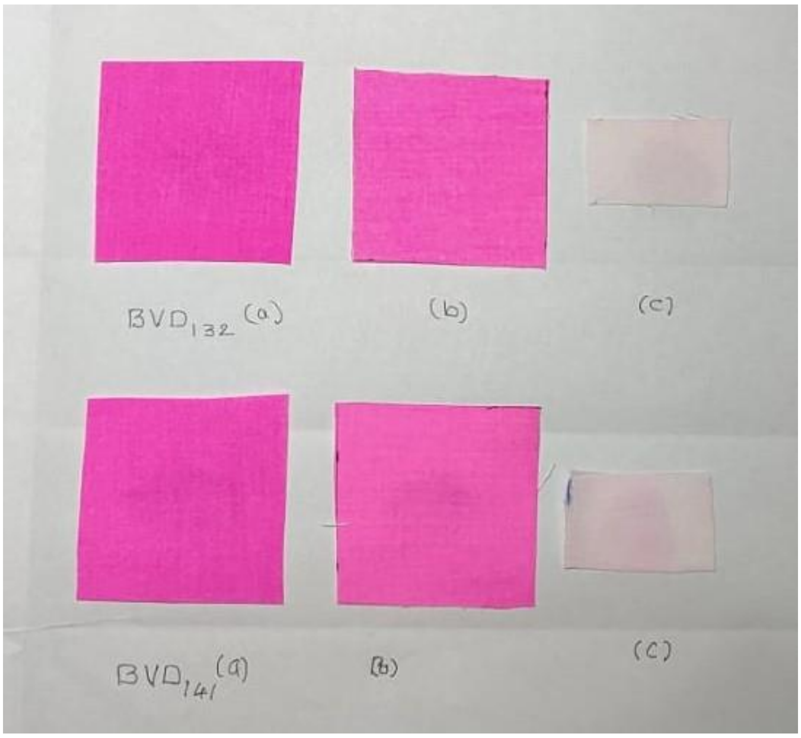

Figure S1. (a) printed fabrics of fluorescent dispersions of BV_{132} and BV_{141} (b) corresponding wash fastness (c) corresponding colour migration of printed fabrics to unprinted fabrics.

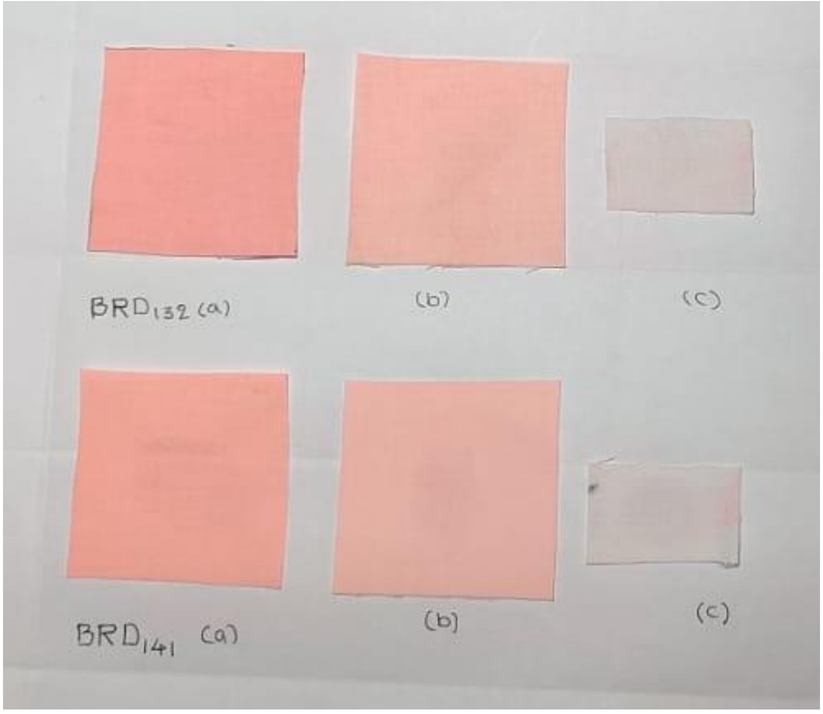

Figure S2. (a) printed fabrics of fluorescent dispersions of BRD132 and BRD141 (b) corresponding wash fastness (c) corresponding colour migration of printed fabrics to unprinted fabrics.

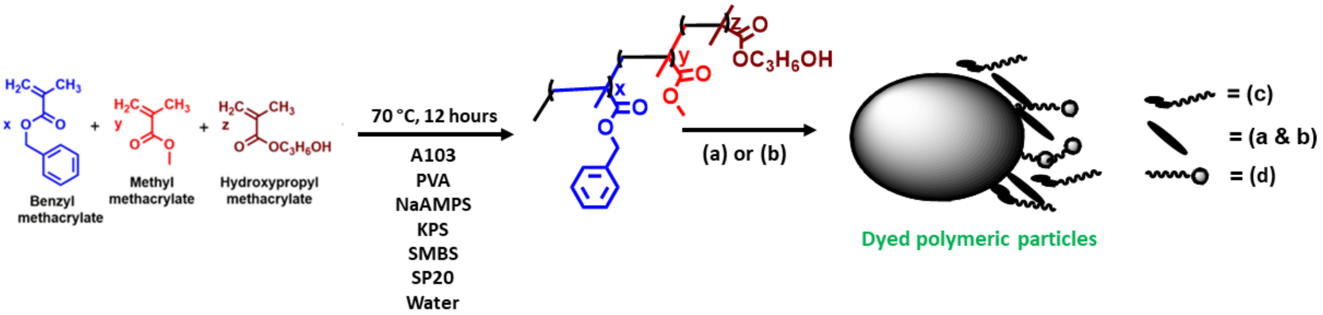

Figure S3. Schematic representation of a series of acrylic nanodispersions were synthesized by varying the moles ratio of benzyl methacrylate (BZMA), methyl methacrylate (MMA), and 2-hydroxypropyl methacrylate (HPMA) monomers. (a) Basic Red 1:1, (b) Basic Violet 11:1, (c) Styrenated phenol ethoxylate, and (d) A103 surfactant.
